# Development and validation of nomograms for predicting the prognosis of early and late recurrence of advanced gastric cancer after radical surgery based on post-recurrence survival

**DOI:** 10.1097/MD.0000000000038376

**Published:** 2024-05-31

**Authors:** Chenming Liu, Jialiang Lu, Liang An

**Affiliations:** a Department of General Surgery, Shaoxing People’s Hospital, Shaoxing, China; b Zhejiang University School of Medicine, Hangzhou, China; c School of Medicine, Shaoxing University, Shaoxing, China; d Department of Gastrointestinal Surgery, Shaoxing People’s Hospital, Shaoxing, China.

**Keywords:** gastrectomy, gastric cancer, nomogram, prediction, recurrence

## Abstract

In this study, we aimed to explore the risk factors influencing post-recurrence survival (PRS) of early recurrence (ER) and late recurrence (LR) in stage advanced gastric cancer (AGC) patients after radical surgery, respectively, and to develop predictive models in turn. Medical records of 192 AGC patients who recurred after radical gastrectomy were retrospectively reviewed. They were randomly divided into the training and validation set at a ratio of 2:1. Nomograms were built based on risk factors influencing PRS of ER and LR explored by Cox regression analyses, respectively. Concordance index (C-index) values and calibration curves were used to evaluate predictive power of nomograms. Body mass index < 18.5 kg/m^2^, prealbumin level < 70.1 mg/L, positive lymph nodes ratio ≥ 0.486 and palliative treatment after recurrence were independent risk factors for the prognosis of ER. In contrast, prealbumin level < 170.1 mg/L, CEA ≥ 18.32 μg/L, tumor diameter ≥ 5.5 cm and palliative treatment after recurrence were independent risk factors for the prognosis of LR. The C-index values were 0.801 and 0.772 for ER and LR in the training set, respectively. The calibration curves of validation set showed a C-index value of 0.744 and 0.676 for ER and LR, respectively. Nomograms which were constructed to predict the prognosis of ER and LR of AGC after surgery showed great predictive power and could provide reference for clinicians’ treatment strategies to some extent.

## 1. Introduction

Gastric cancer (GC) is the sixth most common malignancy and the third leading cause of cancer-related death worldwide.^[1]^ Although a variety of treatment methods have been gradually introduced into clinical practice in recent years, the prognosis of GC is still unsatisfactory and its 5-year survival is no more than 20%.^[2]^ Radical gastrectomy is still the mainstream treatment strategy for patients with GC.^[3]^ More than half of GC patients experience recurrence within 2 years after surgery.^[4,5]^ Therefore, it is of great use to actively explore the prediction model accurately.

Previous studies have well elucidated the predictive role of certain tumor-specific factors during the recurrence of GC, especially early recurrence (ER), such as degree of tumor differentiation, tumor size, and perineural invasion.^[6–8]^ Furthermore, predictive models constructed based on these risk factors showed good predictive performance in practical clinical setting.^[9,10]^ But these studies included only patients with early GC or combined patients with early and advanced GC. AGC tends to have completely different characteristics from early GC due to its greater likeliness of invasion and metastasis. Predictors of prognosis after late recurrence (LR) of GC are not fully understood.

The objective of this study is to explore the risk factors influencing the prognosis of ER and LR in AGC patients after radical surgery, respectively, and to build predictive models in turn, in order to timely spot high-risk patients and implement early intervention.

## 2. Methods

### 2.1. Study population

**Figure 1.**
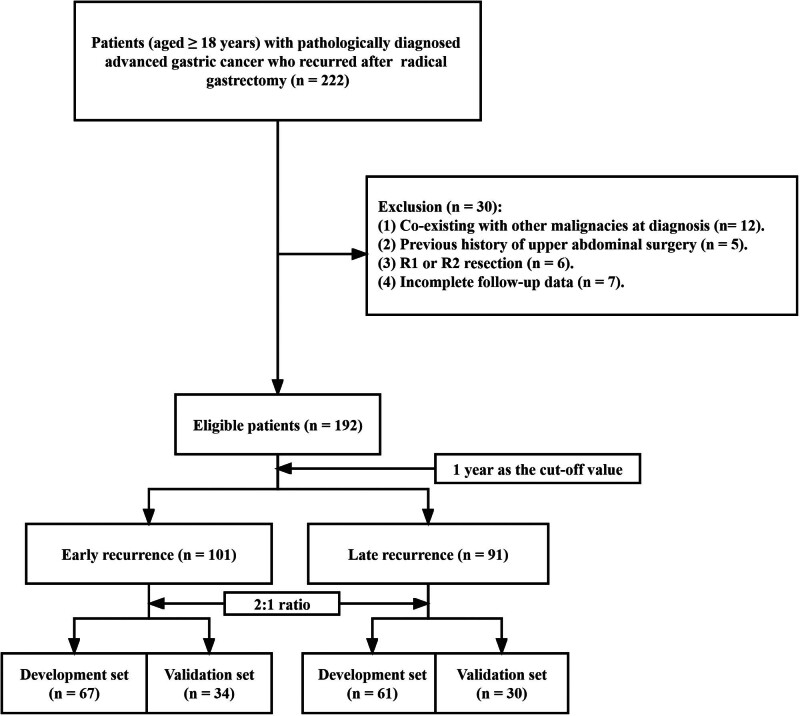
The flowchart of study population.

Medical records of patients with pathologically diagnosed AGC who underwent radical gastrectomy at our institution from 2016 to 2020 were retrospectively collected. Inclusion criteria were as follows: age over 18 years at diagnosis; neoadjuvant therapy before surgery was not performed; recurrence was diagnosed by imaging or pathological examination after surgery. Patients who met the following criteria were excluded: co-existing with other malignancies at diagnosis; previous history of upper abdominal surgery; R1 or R2 resection; incomplete follow-up data. This study was approved by the Ethics Committee of our Institute and met the criteria of the Declaration of Helsinki.^[11]^ Random numbers generated by the computer divided the enrolled patients into the training set and the validation set in a 2:1 ratio. According to the latest surgical guidelines for GC in Japan, gastrectomy and D2 lymph node dissection were performed.^[12]^ The TNM staging of the tumor was based on the 8th edition of the American Joint Committee on Cancer staging system.^[13]^ Fluorouracil and platinum-based regimen (usually 3-weel cycles of capecitabine/S-1 and oxaliplatin) was recommended for chemotherapy after surgery, depending on the patient physical condition and willingness.^[14,15]^ The detailed flow chart is shown in Figure 1.

### 2.2. Variables

Clinicopathological variables included demographic information, surgery-related variables, pathology-related variables, and relevant nutritional and inflammatory variables at and after recurrence. As previously reported, prognostic nutritional index (PNI) was assessed using the following formula: PNI = serum albumin level (g/L) + 0.005 × peripheral blood total lymphocyte count (per mm^3^).^[16]^ The positive lymph node ratio (PLNR) was calculated as the total number of metastatic lymph nodes/total number of lymph nodes. In order to maintain the objectivity of the data, the receiver operating characteristic (ROC) curve was used to determine the optimal cutoff value for most continuous variables, such as, age and albumin. For detailed information, the additional material was shown (Table S1, http://links.lww.com/MD/M690 S2, http://links.lww.com/MD/M691). For body mass index (BMI) and anemia, widely accepted definitions were used.

### 2.3. Definition

The cutoff value for the discrimination of ER and LR was defined as 1 year, based on criteria generally accepted in previous studies.^[10,17]^ Post-recurrence survival (PRS) was defined as the time interval between initial diagnosis of tumor recurrence and death or 3 years after recurrence. Recurrence encompassed loco-regional, peritoneal, and distant lymph node recurrence, as well as single-organ recurrence and multiple organ recurrence. Loco-regional recurrence referred to tumor recurrence in situ or local lymph node recurrence. Organ recurrence included tumor recurrence in other parenchymal organs such as the liver, lung, and bone. Distant lymph node recurrence involved tumor recurrence in regions like the neck, armpit, and subclavian lymph nodes. Multiple organ recurrence refers to the recurrence of 2 or more organs.

### 2.4. Follow-up

Patients were followed up every 2 to 3 months for the first 2 years after discharge and every 6 months thereafter until recurrence. Follow-up included blood tests, chest X-ray, abdominal computed tomography or magnetic resonance imaging, and endoscopic tissue biopsy if necessary. Recurrence diagnosed by regular surveillance was defined as diagnosis by medical imaging and/or specific tumor bio-markers at an interval of 2 to 3 months or less on asymptomatic patients. Recurrence diagnosed by irregular surveillance was defined as those patients having an interval of more than 3 months when recurrences were diagnosed by medical imaging or specific tumor bio-markers, or because examinations were carried out for evident symptoms or other unrelated reasons.^[18]^ Post-recurrence treatment included palliative treatment, adjuvant chemotherapy and re-surgical intervention, which were selected according to the practical condition and willingness of patients.

### 2.5. Statistical analysis

For continuous variables distributed normally in the whole cohort, the mean and standard deviation were calculated, and a t-test was used to assess differences between groups. Otherwise, the median and the interquartile range were calculated, and compared using a Wilcoxon test. Categorical variables were described as frequency (%) and analyzed using a chi-square test or Fisher exact test. Kaplan–Meier method and log-rank test were used for survival analysis. Multivariate Cox regression analysis was used to determine the independent risk factors for PRS with ER and LR respectively. Nomograms were constructed based on these risk factors. The data sets were divided into 2 groups: the larger data set was used to develop the model, and the smaller data set was used to validate the built model. The model predictions were evaluated according to the concordance index (C-index) of 0-1 and 95% confidence interval (CI) and the area under the curve (AUC) of the ROC curve. The bootstrap method (frequency = 1000) was used for internal validation, the C-index was calculated and the calibration curve was drawn. Then, decision curve analysis (DCA) of the nomogram was calculated. All statistical analysis and data processing were performed SPSS software (version 25.0, IBM Corp., Armonk, NY) and R software (version 4.1.2; R Foundation for Statistical Computing). All tests were bilateral, and *P* < .05 was considered significant.

## 3. Results

### 3.1. Characteristics of clinicopathological variables

Among the patients with ER, 73.3% were male, 80.2% were pTNM stage III, and 78.2% received postoperative adjuvant chemotherapy. For the recurrence site, single-organ recurrence was the most common (32.7%). For post-recurrence treatment, 42.6% received chemotherapy and 12.9% received re-surgery intervention. In contrast, for patients with LR, 76.9% were male, 70.3% were pTNM stage III, 87.9% received postoperative adjuvant chemotherapy, and for the recurrence site, distant lymph node recurrence, multiple organs recurrence and peritoneal recurrence were most common (33.0%). For post-recurrence treatment, 49.5% received chemotherapy and 20.9% received re-surgery intervention. The detailed clinicopathological characteristics of ER and LR are shown in Tables 1 and 2, respectively. No obvious differences were found between training and validation sets (all *P* > .05).

**Table 1:** Comparison of characteristics between training and validation cohort in patients with early recurrence.

Variables	Training set (N = 67)	Validation set (N = 34)	*P*
Sex			.399
Male	48 (71.6%)	27 (79.4%)	
Female	19 (28.4%)	7 (20.6%)	
Age (yr)			.522
< 68	29 (43.3%)	17 (50.0%)	
≥ 68	38 (56.7%)	17 (50.0%)	
Smoking			.578
Yes	20 (29.9%)	12 (35.3%)	
No	47 (70.1%)	22 (64.7%)	
Drinking			.905
Yes	17 (25.4%)	9 (26.5%)	
No	50 (74.6%)	25 (73.5%)	
FOBT			.843
Positive	23 (34.3%)	11 (32.4%)	
Negative	44 (65.7%)	23 (67.6%)	
BMI (kg/m^2^)			.961
Low	22 (32.8%)	11 (32.4%)	
Normal	45 (67.2%)	23 (67.6%)	
PNI			.966
<35.65	18 (26.9%)	9 (26.5%)	
≥35.65	49 (73.1%)	25 (73.5%)	
Hb (g/L)			.964
Low	47 (70.1%)	24 (70.6%)	
Normal	20 (29.9%)	10 (29.4%)	
ALB (g/L)			.841
<32.5	27 (40.3%)	13 (38.2%)	
≥32.5	40 (59.7%)	21 (61.8%)	
PAB (mg/L)			.459
<70.1	17 (25.4%)	11 (32.4%)	
≥70.1	50 (74.6%)	23 (67.6%)	
NLR			.956
<2.018	26 (38.8%)	13 (38.2%)	
≥2.018	41 (61.2%)	21 (61.8%)	
LCR			.842
<0.535	46 (68.7%)	24 (70.6%)	
≥0.535	21 (31.3%)	10 (29.4%)	
PLR			.982
<71.681	6 (9.0%)	3 (8.8%)	
≥71.681	61 (91.0%)	31 (91.2%)	
LMR			.619
<2.286	30 (44.8%)	17 (50.0%)	
≥2.286	37 (55.2%)	17 (50.0%)	
CEA (μg/L)			.346
<4.4	25 (37.3%)	16 (47.1%)	
≥4.4	42 (62.7%)	18 (52.9%)	
CA199 (KU/L)			.363
< 25.52	35 (52.2%)	21 (61.8%)	
≥ 25.52	32 (47.8%)	13 (38.2%)	
Recurrence pattern			.892
Loco-regional	2 (3.0%)	1 (2.9%)	
Distant lymph nodes	9 (13.4%)	3 (8.8%)	
Peritoneal	19 (28.4%)	10 (29.4%)	
Single organ	20 (29.9%)	13 (38.2%)	
Multiple organs	17 (25.4%)	7 (20.6%)	
Resection range			.440
Total	29 (43.3%)	12 (35.3%)	
Subtotal	38 (56.7%)	22 (64.7%)	
Surgical method			.755
Billroth-I	4 (6.0%)	3 (8.8%)	
Billroth-I	35 (52.2%)	19 (55.9%)	
Roux-en-Y	28 (41.8%)	12 (35.3%)	
Surgical procedure			.078
Open	40 (59.7%)	14 (41.2%)	
Laparoscopic	27 (40.3%)	20 (58.8%)	
Adjuvant chemotherapy			.762
Yes	53 (79.1%)	26 (76.5%)	
No	14 (20.9%)	8 (23.5%)	
PLNR			.840
<0.486	38 (56.7%)	20 (58.8%)	
≥0.486	29 (43.3%)	14 (41.2%)	
Differentiation degree			.276
Moderate	7 (10.4%)	7 (20.6%)	
Poor	60 (89.6%)	27 (79.4%)	
Signet-ring cell carcinoma			.197
Yes	15 (22.4%)	4 (11.8%)	
No	52 (77.6%)	30 (88.2%)	
Tumor size (cm)			.964
<4.4	20 (29.9%)	10 (29.4%)	
≥4.4	47 (70.1%)	24 (70.6%)	
Tumor location			.525
High	8 (11.9%)	2 (5.9%)	
Middle	15 (22.4%)	10 (29.4%)	
Low	44 (65.7%)	22 (64.7%)	
Borrmann classification			.522
I	3 (4.5%)	1 (2.9%)	
II	0 (0%)	0 (0%)	
III	53 (79.1%)	30 (88.2%)	
IV	11 (16.4%)	3 (8.8%)	
pTNM			.503
II	12 (17.9%)	8 (23.5%)	
III	55 (82.1%)	26 (76.5%)	
Invasion area			.586
None	28 (41.8%)	16 (47.1%)	
Vascular	11 (16.4%)	7 (20.6%)	
Perineural	10 (14.9%)	6 (17.6%)	
Vascular and perinural	18 (26.9%)	5 (14.7%)	
Post-recurrence treatment			.152
Support	33 (49.3%)	12 (35.3%)	
Chemotherapy	24 (35.8%)	19 (55.9%)	
Surgery	10 (14.9%)	3 (8.8%)	
Postoperative surveillance			.947
Irregular	35 (52.2%)	18 (52.9%)	
Regular	32 (47.8%)	16 (47.1%)	

ALB = albumin, BMI = body mass index, CA 199 = carbohydrate antigen 199, CEA = carcinoembryonic antigen, FOBT = focal occult blood test, Hb = hemoglobin, LCR = lymphocyte-to-C-reactive ratio, LMR = lymphocyte-to-monocyte ratio, NLR = neutrophil-to-lymphocyte ratio, PAB = prealbumin, PLNR = positive lymph nodes ratio, PLR = platelet-to-lymphocyte ratio, PNI = prognostic nutrition index, pTNM = pathologic tumor node metastasis.

**Table 2:** Comparison of characteristics between training and validation cohort in patients with late recurrence.

Variables	Training set (N = 61)	Validation set (N = 30)	*P*
Sex			.569
Male	48 (78.7%)	22 (73.3%)	
Female	13 (21.3%)	8 (26.7%)	
Age (yr)			.215
<72	48 (78.7%)	20 (66.7%)	
≥72	13 (21.3%)	10 (33.3%)	
Smoking			.130
Yes	24 (39.3%)	7 (23.3%)	
No	37 (60.7%)	23 (76.7%)	
Drinking			.353
Yes	20 (32.8%)	7 (23.3%)	
No	41 (67.2%)	23 (76.7%)	
FOBT			.067
Positive	10 (16.4%)	10 (33.3%)	
Negative	51 (83.6%)	20 (66.7%)	
BMI (kg/m^2^)			.113
Low	20 (32.8%)	15 (50.0%)	
Normal	41 (67.2%)	15 (50.0%)	
PNI			.923
<44	38 (62.3%)	19 (63.3)	
≥44	23 (37.7%)	11 (36.7)	
Hb (g/L)			.811
Low	35 (57.4%)	18 (60.0)	
Normal	26 (42.6%)	12 (40.0)	
ALB (g/L)			.955
<37.7	39 (63.9%)	19 (63.3)	
≥37.7	22 (36.1%)	11 (36.7)	
PAB (mg/L)			.603
<170.1	32 (52.5%)	14 (46.7%)	
≥170.1	29 (47.5%)	16 (53.3%)	
NLR			.852
<2.748	29 (47.5%)	15 (50.0%)	
≥2.748	32 (52.5%)	15 (50.0%)	
LCR			.830
<1.729	46 (75.4%)	22 (73.3%)	
≥1.729	15 (24.6%)	8 (26.7%)	
PLR			.158
<156.618	34 (55.7%)	12 (40.0%)	
≥156.618	27 (44.3%)	18 (60.0%)	
LMR			.084
<3.073	40 (65.6%)	14 (46.7%)	
≥3.073	21 (34.4%)	16 (53.3%)	
CEA (μg/L)			.551
<18.32	50 (82.0%)	23 (76.7%)	
≥18.32	11 (18.0%)	7 (23.3%)	
CA199 (KU/L)			1.000
<3.73	10 (16.4%)	5 (16.7%)	
≥3.73	51 (83.6%)	25 (83.3%)	
Recurrence pattern			.979
Loco-regional	5 (8.2%)	2 (6.7%)	
Distant lymph nodes	8 (13.1%)	3 (10.0%)	
Peritoneal	19 (31.1%)	11 (36.7%)	
Single organ	20 (32.8%)	10 (33.3%)	
Multiple organs	9 (14.8%)	4 (13.3%)	
Resection range			.503
Total	22 (36.1%)	13 (43.3%)	
Subtotal	39 (63.9%)	17 (56.7%)	
Surgical method			.615
Billroth-I	9 (14.8%)	5 (16.7%)	
Billroth-I	31 (50.8%)	12 (40.0%)	
Roux-en-Y	21 (34.4%)	13 (43.3%)	
Surgical procedure			.606
Open	27 (44.3%)	15 (50.0%)	
Laparoscopic	34 (55.7%)	15 (50.0%)	
Adjuvant chemotherapy			.550
Yes	55 (90.2%)	25 (83.3%)	
No	6 (9.8%)	5 (16.7%)	
PLNR			.152
<0.289	31 (50.8%)	20 (66.7%)	
≥0.289	30 (49.2%)	10 (33.3%)	
Differentiation degree			.304
Well	0 (0%)	1 (3.3%)	
Moderate	9 (14.8%)	3 (10.0%)	
Poor	52 (85.2%)	26 (86.7%)	
Signet-ring cell carcinoma			.204
Yes	20 (32.8%)	6 (20.0%)	
No	41 (67.2%)	24 (80.0%)	
Tumor size (cm)			.326
<5.5	32 (52.5%)	19 (63.3%)	
≥5.5	29 (47.5%)	11 (36.7%)	
Tumor location			.583
High	9 (14.8%)	7 (23.3%)	
Middle	15 (24.6%)	6 (20.0)	
Low	37 (60.7%)	17 (56.7%)	
Borrmann classification			.501
I	2 (3.3%)	0 (0%)	
II	1 (1.6%)	1 (3.3%)	
III	54 (88.5%)	25 (83.3%)	
IV	4 (6.6%)	4 (13.3%)	
pTNM			.306
II	16 (26.2%)	11 (36.7%)	
III	45 (73.8%)	19 (63.3%)	
Invasion area			.716
None	26 (42.6%)	12 (40.0%)	
Vascular	9 (14.8%)	7 (23.3%)	
Perineural	19 (31.1%)	9 (30.0%)	
Vascular and perinural	7 (11.5%)	2 (6.7%)	
Post-recurrence treatment			.990
Support	18 (29.5%)	9 (30.0%)	
Chemotherapy	30 (49.2%)	15 (50.0%)	
Surgery	13 (21.3%)	6 (20.0%)	
Postoperative surveillance			.673
Irregular	40 (65.6%)	21 (70.0%)	
Regular	21 (34.4%)	9 (30.0%)	

ALB = albumin, BMI = body mass index, CA 199 = carbohydrate antigen 199, CEA = carcinoembryonic antigen, FOBT = focal occult blood test, Hb = hemoglobin, LCR = lymphocyte-to-C-reactive ratio, LMR = lymphocyte-to-monocyte ratio, NLR = neutrophil-to-lymphocyte ratio, PAB = prealbumin, PLNR = positive lymph nodes ratio, PLR = platelet-to-lymphocyte ratio, PNI = prognostic nutrition index, pTNM = pathologic tumor node metastasis.

### 3.2. Comparison of recurrence patterns

For patients with ER, the most common recurrence pattern was single-organ recurrence (29.9%), followed by peritoneal recurrence (28.4%). To be slightly different, for patients with LR, the most common recurrence pattern was peritoneal recurrence (32.8%), followed by distant lymph node recurrence and multiple organs recurrence (31.3%). The recurrence patterns were similar between the 2 groups (all *P* > .05) (Table 3).

**Table 3:** Recurrence patterns between early and late recurrence.

Recurrence pattern	Early recurrence (n = 67)	Late recurrence (n = 61)	*P*
Loco-regional	2 (3.0%)	5 (8.2%)	.365
Distant lymph nodes	9 (13.4%)	8 (13.1%)	.958
Peritoneal	19 (28.4%)	19 (31.1%)	.730
Single organ	20 (29.9%)	20 (32.8%)	.720
Multiple organs	17 (25.4%)	9 (14.8%)	.136

### 3.3. Multivariate Cox regression analysis of patients with ER and LR

Multivariate Cox regression analysis showed that BMI < 18.5 kg/m^2^, prealbumin level < 70.1 mg/L, PLNR ≥ 0.486 and palliative treatment after recurrence were independent risk factors for the prognosis of ER (Table 4). In contrast, prealbumin level < 170.1 mg/l at recurrence, CEA ≥ 18.32 μg/L, tumor diameter ≥ 5.5 cm and palliative treatment after recurrence were independent risk factors for prognosis of LR (Table 5).

**Table 4:** Univariate and multivariate Cox regression analysis of PRS in gastric cancer with early recurrence.

Characteristics	Univariate analysis		Multivariate analysis	
	HR (95% CI)	*P*	HR (95% CI)	*P*
Sex, female vs male	1.334 (0.763–2.332)	.313		
Age (yr), ≥ 68 vs < 68	1.270 (0.755–2.137)	.368		
Smoking, yes vs no	1.571 (0.913–2.703)	.103		
Drinking, yes vs no	1.180 (0.663–2.098)	.574		
FOBT, positive vs negative	1.118 (0.651–1.919)	.687		
BMI (kg/m^2^), normal vs low	0.335 (0.193–0.581)	<.001^*^	0.551 (0.303–0.999)	.049^*^
PNI, ≥ 35.65 vs < 35.65	0.358 (0.201–0.638)	<.001^*^		
Hb (g/L), normal vs low	0.904 (0.515–1.589)	.727		
ALB (g/L), ≥ 32.5 vs < 32.5	0.610 (0.363–1.026)	.062		
PAB (mg/L), ≥ 70.1 vs < 70.1	0.326 (0.182–0.586)	<.001^*^	0.289 (0.150–0.558)	<.001^*^
NLR, ≥ 2.018 vs < 2.018	2.822 (1.619–4.916)	<.001^*^		
LCR, ≥ 0.535 vs < 0.535	0.525 (0.298–0.927)	.026^*^		
PLR, ≥ 71.681 vs < 71.681	3.304 (1.027–10.628)	.045^*^		
LMR, ≥ 2.286 vs < 2.286	0.507 (0.302–0.850)	.010^*^		
CEA (μg/L), ≥ 4.4 vs < 4.4	1.443 (0.833–2.502)	.191		
CA199 (KU/L), ≥ 25.52 vs < 25.52	1.820 (1.081–3.062)	.024^*^		
Recurrence pattern	Ref - loco-regional			
Distant lymph nodes	2.490 (0.305–20.307)	.394		
Peritoneal	3.017 (0.401–22.712)	.284		
Single organ	5.015 (0.667–37.684)	.117		
Multiple organs	5.553 (0.728–42.391)	.098		
Resection range, Subtotal vs total	0.979 (0.585–1.638)	.937		
Surgical method	Ref - Billroth-I			
Billroth-II	1.612 (0.489–5.318)	.433		
Roux-en-Y	1.473 (0.442–4.909)	.528		
Operative type, LAP vs OP	0.624 (0.366–1.063)	.082		
Adjuvant chemotherapy, yes or no	1.027 (0.545–1.938)	.933		
PLNR, ≥ 0.486 vs < 0.486	2.842 (1.626–4.907)	<.001^*^	2.879 (1.607–5.159)	<.001^*^
Differentiation, poor vs moderate	0.948 (0.407–2.211)	.902		
Signet-ring cell carcinoma, yes vs no	0.947 (0.510–1.757)	.862		
Tumor size (cm), ≥ 4.4 vs < 4.4	1.418 (0.795–2.530)	.237		
Tumor location	Ref- High			
Middle	1.265 (0.497–3.221)	.622		
Low	1.729 (0.771–3.877)	.184		
Borrmann classification	Ref- I			
III	2.517 (0.609–10.395)	.202		
IV	4.117 (0.891–19.017)	.070		
pTNM, III vs II	1.674 (0.843–3.325)	.141		
Invasion area	Ref - none			
Peri-vascular	0.960 (0.458–2.011)	.914		
Perineural	1.113 (0.500–2.481)	.793		
Peri-vascular and neural	1.920 (1.016–3.631)	.045		
Post-recurrence treatment	Ref - palliative			
Adjuvant chemotherapy	0.456 (0.260–0.801)	.006^*^	0.402 (0.214–0.755)	.005^*^
Re-surgery intervention	0.332 (0.144–0.766)	.010^*^	0.398 (0.171–0.925)	.032^*^
Postoperative surveillance, no vs yes	2.184 (1.296–3.681)	.003^*^		

ALB = albumin, BMI = body mass index, CA 199 = carbohydrate antigen 199, CEA = carcinoembryonic antigen, FOBT = focal occult blood test, Hb = hemoglobin, LAP = laparoscopic, LCR = lymphocyte-to-C-reactive ratio, LMR = lymphocyte-to-monocyte ratio, NLR = neutrophil-to-lymphocyte ratio, OP = open, PAB = prealbumin, PLNR = positive lymph nodes ratio, PLR = platelet-to-lymphocyte ratio, PNI = prognostic nutrition index, pTNM = pathologic tumor node metastasis.

**Table 5:** Univariate and multivariate Cox regression analysis of PRS in gastric cancer with late recurrence.

Characteristics	Univariate analysis		Multivariate analysis	
	HR (95 CI%)	*P*	HR (95 CI%)	*P*
Sex, female vs male	1.618 (0.855–3.062)	.140		
Age (yr), ≥ 72 vs < 72	1.638 (0.871–3.080)	.126		
Smoking, yes vs no	0.849 (0.487–1.483)	.566		
Drinking, yes vs no	1.014 (0.569–1.807)	.963		
FOBT, positive vs negative	1.556 (0.776–3.120)	.213		
BMI (kg/m^2^), normal vs low	0.404 (0.228–0.715)	.002^*^		
PNI, ≥ 44 vs < 44	0.454 (0.252–0.817)	.008^*^		
Hb (g/L), normal vs low	0.472 (0.269–0.827)	.009^*^		
ALB (g/L), ≥ 37.7 vs < 37.7	0.391 (0.215–0.711)	.002^*^		
PAB (mg/L), ≥ 170.1 vs < 170.1	0.354 (0.202–0.620)	<.001^*^	0.426 (0.239–0.762)	.004^*^
NLR, ≥ 2.748 vs < 2.748	1.779 (1.021–3.101)	.042^*^		
LCR, ≥ 1.729 vs < 1.729	0.492 (0.252–0.959)	.037^*^		
PLR, ≥ 156.618 vs < 156.618	1.493 (0.864–2.578)	.151		
LMR, ≥ 3.073 vs < 3.073	0.669 (0.374–1.199)	.177		
CEA (μg/L), ≥ 18.32 vs < 18.32	2.469 (1.207–5.050)	.013^*^	2.923 (1.369–6.240)	.006^*^
CA199 (KU/L), ≥ 3.73 vs < 3.73	1.869 (0.841–4.150)	.125		
Recurrence pattern	Ref - loco-regional			
Distant lymph nodes	0.416 (0.111–1.558)	.193		
Peritoneal	1.263 (0.424–3.762)	.675		
Single organ	1.080 (0.365–3.200)	.889		
Multiple organs	1.813 (0.551–5.969)	.328		
Resection range, Subtotal vs total	0.861 (0.493–1.505)	.600		
Surgical method	Ref- Billroth-I			
Billroth-II	1.327 (0.577–3.055)	.505		
Roux-en-Y	1.468 (0.615–3.504)	.387		
Operative type, LAP vs OP	1.101 (0.640–1.894)	.728		
Adjuvant chemotherapy, yes or no	1.303 (0.512–3.312)	.578		
PLNR, ≥ 0.289 vs < 0.289	1.434 (0.833–2.467)	.194		
Differentiation, poor vs moderate	1.189 (0.536–2.641)	.670		
Signet-ring cell carcinoma, yes vs no	1.056 (0.597–1.870)	.851		
Tumor size (cm), ≥ 5.5 vs < 5.5	1.964 (1.131–3.413)	.017^*^	2.497 (1.388–4.491)	.002^*^
Tumor location	Ref- high			
Middle	0.785 (0.324–1.902)	.592		
Low	0.898 (0.412–1.958)	.787		
Borrmann classification	Ref- I			
II	0.413 (0.037–4.671)	.475		
III	0.313 (0.073–1.348)	.119		
IV	0.801 (0.144–4.465)	.800		
pTNM, III vs II	1.152 (0.625–2.122)	.651		
Invasion area	Ref - none			
Peri-vascular	1.719 (0.782–3.778)	.177		
Perineural	0.845 (0.442–1.613)	.609		
Peri-vascular and neural	1.044 (0.421–2.587)	.926		
Post-recurrence treatment	Ref - palliative			
Adjuvant chemotherapy	0.376 (0.197–0.715)	.003^*^	0.328 (0.159–0.674)	.002^*^
Re-surgery intervention	0.395 (0.180–0.866)	.020^*^	0.420 (0.184–0.955)	.039^*^
Postoperative surveillance, no vs yes	2.160 (1.194–3.907)	.011^*^		

ALB = albumin, CA 199 = carbohydrate antigen 199, CEA = carcinoembryonic antigen, FOBT = focal occult blood test; BMI, body mass index, Hb = hemoglobin, LAP = laparoscopic, LCR = lymphocyte-to-C-reactive ratio, LMR = lymphocyte-to-monocyte ratio, NLR = neutrophil-to-lymphocyte ratio, OP = open, PAB = prealbumin, PLNR = positive lymph nodes ratio, PLR = platelet-to-lymphocyte ratio, PNI = prognostic nutrition index, pTNM = pathologic tumor node metastasis.

### 3.4. PRS for patients with ER and LR

**Figure 2.**
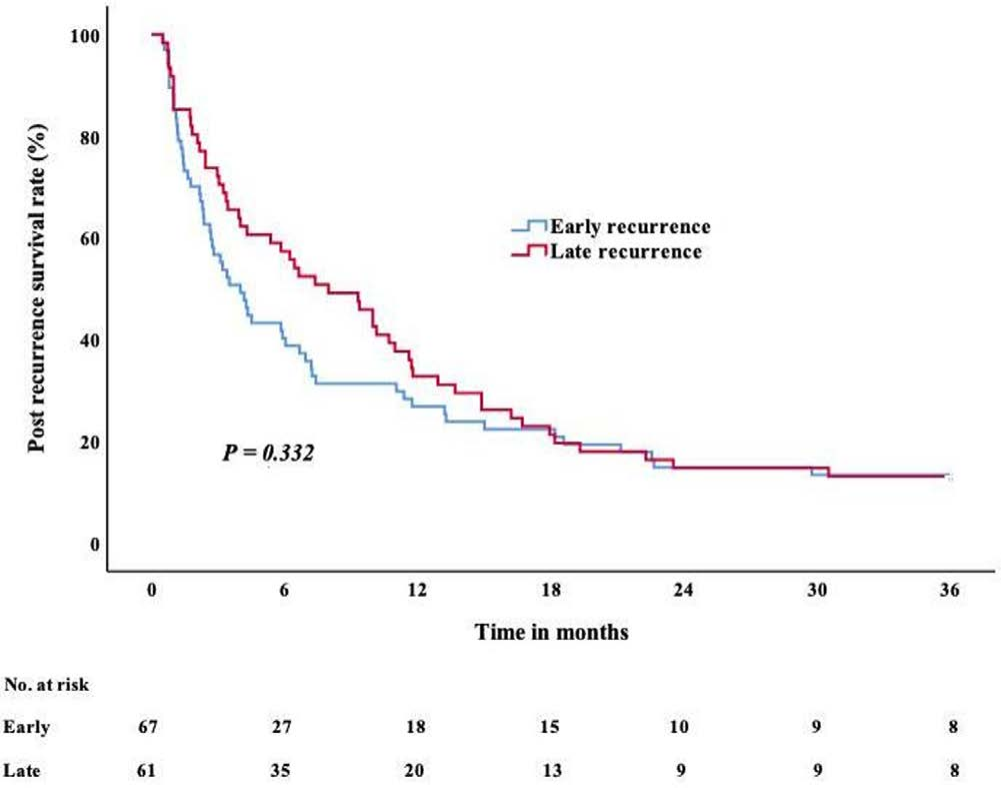
Kaplan–Meier curve of PRS for ER and LR. ER = early recurrence, LR = late recurrence, PRS = post-recurrence survival.

Kaplan–Meier curves showed no significant difference in median PRS between patients with ER and LR (Fig. 2). Subgroup survival analyses were then performed on the basis of prealbumin level and categories of post-recurrence treatment. The results showed that lower prealbumin level at recurrence had worse prognosis than higher prealbumin level (ER: 1 vs 6 months, *P *< .001; LR: 3 vs 13 months, *P *< .001) (Figure S1, http://links.lww.com/MD/M692, S2, http://links.lww.com/MD/M693). Compared with re-surgery intervention and adjuvant chemo-radiotherapy, patients who received palliative treatment after recurrence had worse PRS (for ER, 2 vs 7 vs 7 months, *P *=* *.003; for LR, 2 vs 13 vs 11 months, *P *= .005) (Figure S3, http://links.lww.com/MD/M694 S4, http://links.lww.com/MD/M695).

### 3.5. Development and validation of nomograms

**Figure 3.**
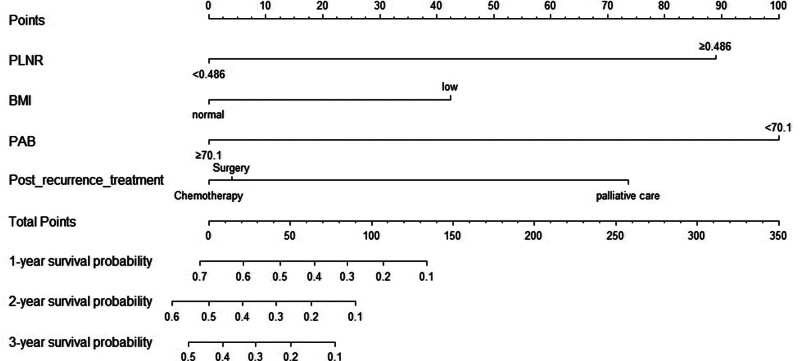
Nomogram including risk factors of PRS for ER. ER = early recurrence, PRS = post-recurrence survival.

**Figure 4.**
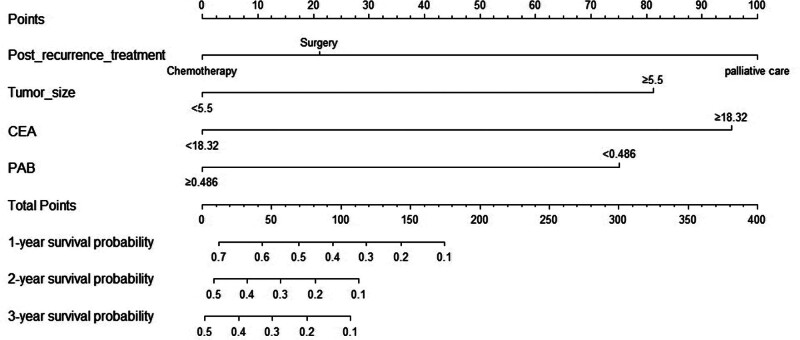
Nomogram including risk factors of PRS for LR. LR = late recurrence, PRS = post-recurrence survival.

For patients with ER and LR, nomograms were constructed on the basis of these 4 independent predictors in the training set, respectively (Figs. 3 and 4).

**Figure 5.**
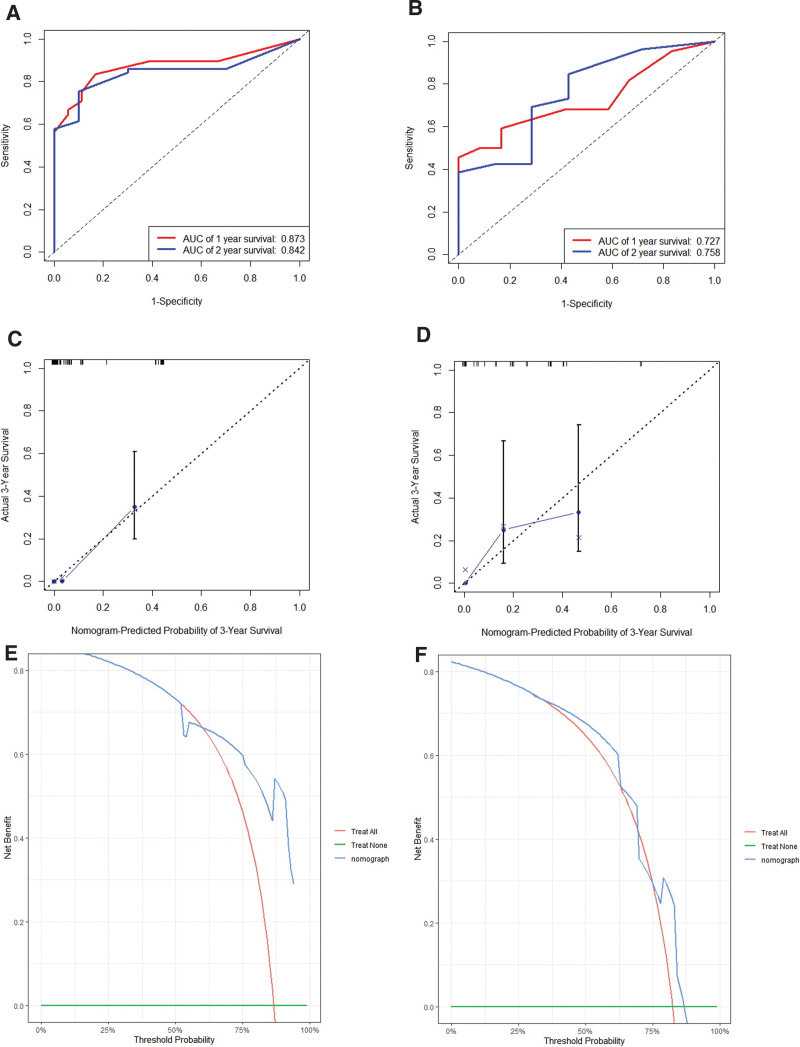
Receiver operating characteristics (A, training set; B, validation set), calibration curves (C, training set; D, validation set) and decision curves (E, training set; F, validation set) for predicting PRS of ER. ER = early recurrence, PRS = post-recurrence survival.

For ER, the training and validation set showed that the C-index was 0.801 (95% CI, 0.746–0.856) and 0.744 (95% CI, 0.630–0.856), respectively. And the AUC of 1 and 2-year survival was shown in Figure 5A and B. The calibration curves showed a good fit between prediction and actual observation (Fig. 5C and D). Then, the decision curves also demonstrated good predictive power (Fig. 5E and F).

**Figure 6.**
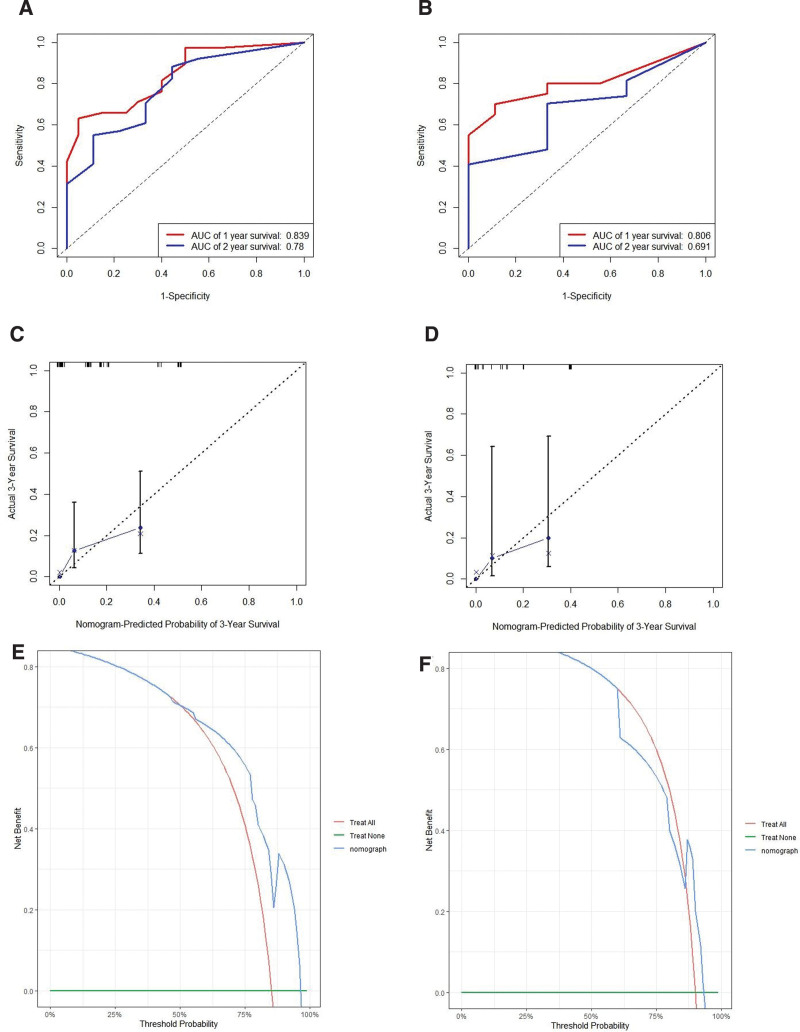
Receiver operating characteristics (A, training set; B, validation set), calibration curves (C, training set; D, validation set) and decision curves (E, training set; F, validation set) for predicting PRS of LR. LR = late recurrence, PRS = post-recurrence survival.

Similarly, for LR, the training and validation set showed that the C-index was 0.772 (95% CI, 0.709–0.835) and 0.676 (95% CI, 0.582–0.770), respectively. And the AUC of 1 and 2 year survival was shown in Figure 6A and B. The calibration curves showed a good fit between prediction and actual observation (Fig. 6C and D). Then, the decision curves also demonstrated good predictive power (Fig. 6E and F).

### 3.6. Exploration of the predictive ability of nomogram

According to different nomogram scores, the predictive probability of ER was divided into 2 risk groups (low-risk group and high-risk group) to further evaluate the predictive power of the nomogram.

**Figure 7.**
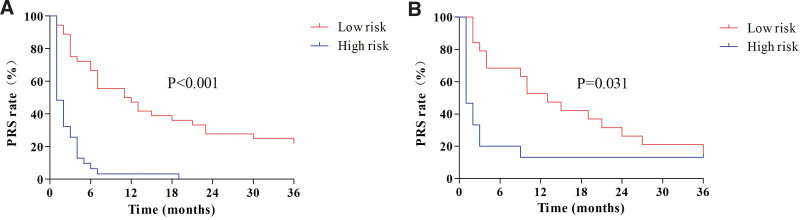
Kaplan–Meier curve of PRS between high-risk and low-risk groups for ER. PRS, post-recurrence survival; ER, early recurrence. (A) Training set; (B) Validation set.

As shown in Figure 7A and B, Kaplan–Meier survival curves showed that the nomogram risk grouping had better discrimination power for PRS in the training and validation set (*P *< .001, *P *= .031). The median PRS in the low-risk group was significantly longer than that in the high-risk group for ER (11 months vs 1 month; 13 months vs 1 month).

**Figure 8.**
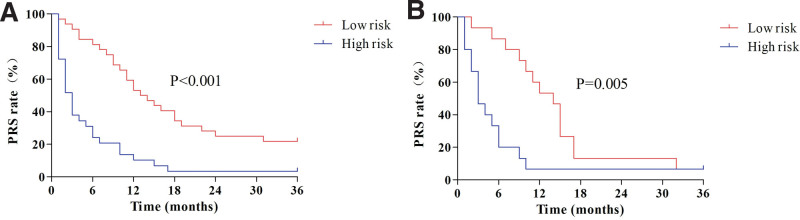
Kaplan–Meier curve of PRS between high-risk and low-risk groups for LR. LR = late recurrence, PRS = post-recurrence survival. (A) Training set; (B) Validation set.

Similarly, for LR, Kaplan–Meier survival curves showed that the nomogram risk grouping had better discrimination power for PRS in the training and validation set (*P *< .001, *P *= .005). The median PRS in the low-risk group was significantly longer than that in the high-risk group (13 months vs 3 months; 14 months vs 3 months) (Fig. 8A and B).

## 4. Discussion

At present, the prognosis of AGC remains unsatisfactory, mainly due to its great possibility of recurrence and metastasis.^[1]^ Recurrence is a common and fatal condition in a variety of malignancies, including GC, and often indicates a poor prognosis.^[5–8]^ To the best of our knowledge, our study is the first to develop and validate nomograms in AGC patients with ER and LR, respectively. Our newly developed nomograms enable surgeons to accurately evaluate the prognosis of GC patients with ER and LR by stratification according to different risk factors, so as to take personalized treatment measures.

In this study, we revealed that there existed some differences in predictive factors of PRS between ER and LR. Low BMI and prealbumin level at the time of recurrence, high PLNR and palliative treatment after recurrence were risk factors for the prognosis of ER. In contrast, low prealbumin and high CEA level at recurrence, large tumor diameter and palliative treatment after recurrence were risk factors for the prognosis of LR.

Ma et al first developed a nomogram for predicting ER of stage II/III GC after surgery based on tumor location, pTNM stage, lymphocyte count, postoperative complications, adjuvant chemotherapy and PLNR. Their C-index value was 0.780 with excellent practicability.^[19]^ Similarly, Liu et al accurately predicted the role of age, Lauren classification, preoperative CA 19-9 level, pathological stage, major pathological response, and postoperative complications in early postoperative recurrence of GC.^[10]^ However, unlike these studies, we focused on the predictive role of indicators at and after recurrence. In the present study, for ER, the C-indexes were 0.801 and 0.744 in the training and validation cohort, respectively; and for LR, the C-indexes were 0.772 and 0.676, respectively. And the fit degree between the actual and predicted curve was in accord, indicating our prediction model had strong prediction power.

We found that high levels of CEA at recurrence and large tumor size were independent risk factors for prognosis after LR. Previous studies have shown that patients with elevated CEA before surgery have an increased risk of recurrence, especially for ER.^[20,21]^ However, CEA levels at the time of recurrence in our study were only confirmed in LR. We hypothesized that this inconsistent phenomenon was related to the inclusion of indicators at 2 different periods, that was, before surgery and at recurrence. However, there is currently a lack of relevant studies to confirm this result.

We observed a strong correlation between low BMI at recurrence and the prognosis of ER, but not in the cohort with LR. Consistent with our study, in a large study conducted in Chinese population to dynamically monitor the association between postoperative BMI and prognosis in GC patients, BMI loss of more than 10% within the first year after surgery was found to be associated with poor prognosis.^[22]^ Therefore, we speculate that for patients of ER, controlling BMI level to an appropriate range is favorable to improve PRS.

In the present study, PLNR ≥ 0.486 was demonstrated an independent risk factor for survival of patients with ER, which was basically consistent with Ma et al, who found that patients with PLNR ≥ 0.335 had higher risk of ER than patients with PLNR ≤ 0.335 with stage II/III GC (OR: 3.37, 95% CI 2.37–4.78, *P* < .001).^[19]^ However, this relationship was not observed in patients with LR. Moreover, Komtasu et al concluded that patients with PLNR ≥ 0.4 had higher rate of node recurrence than those with PLNR < 0.4 in pN3 GC.^[23]^ So we speculate early lymph node recurrence might explain the association between higher PLNR and ER. However, the association of PLNR with the prognosis of GC after recurrence has not been fully confirmed.

We found a high level of prealbumin demonstrated a positive association with survival in GC patients who experienced recurrence by setting the cutoff value of 70.1mg/l for ER and 170.1 mg/l for LR based on ROC curve. This relevance was also observed in previous studies. Shen et al investigated the prognostic significance of pretreatment prealbumin in 731 stage II/III GC patients with the cutoff value of 180 mg/l.^[24]^ They found low prealbumin level was an independent risk factor of overall survival (HR: 1.362, 95% CI, 1.094–1.695, *P* = .006) and disease-free survival (HR: 1.369, 95% CI, 1.099–1.706, *P* = .005). Aoyama et al found that the patients with a prealbumin level < 20 mg/dl had significantly poorer outcomes than those with higher prealbumin levels.^[25]^ The HR for the OS was 2.375 (95% CI, 1.362–4.144). In contrast to prealbumin, a statistical association was not found between albumin and PRS. Consequently, owing to its shorter half-time than albumin, prealbumin is a more sensitive index of nutritional change than albumin.^[26,27]^ Our results suggest that if proper nutritional support before recurrence could be given, a low prealbumin concentration may serve as a modifiable risk factor for prognosis. Large-scale randomized controlled trails should be conducted in the future.

Regarding the post-recurrence treatment strategies, we found that the prognosis of recurrent GC patients with re-surgical intervention and adjuvant chemotherapy was significantly better than that of those with palliative treatment alone. However, no significant difference was observed between re-surgical intervention and adjuvant chemotherapy only in median PRS. Compared with other malignant solid tumors, such as recurrent hepatocellular carcinoma and rectal adenocarcinoma, recurrent GC patients rarely received re-surgical intervention, only 14.9% and 21.3% of ER and LR patients, respectively in our study. Previous studies confirmed that re-surgical resection should be implemented only in selected patients with local recurrence in whom complete resection is possible.^[28–30]^

We have to admit that our study has several limitations. First, our study was a single-center retrospective study with limited data. The cutoff value for distinguishing ER and LR was selected based on previous studies, so there may be some deviation from the real data in this study. Second, the generalizability of our findings may be limited by the lack of external validation. Third, other important clinicopathological factors that have an important impact on the prognosis of recurrence, such as novel genetic markers, were not collected. Despite these limitations, our study is the first to develop predictive models based on independent risk factors for the prognosis of ER and LR of GC. In the future, expect external data from large-scale centers to validate the established models.

## 5. Conclusion

In conclusion, Body mass index < 18.5 kg/m^2^, prealbumin level < 70.1 mg/L, Positive lymph nodes ratio ≥ 0.486 and palliative treatment after recurrence were independent risk factors for the prognosis of ER. In contrast, prealbumin level < 170.1 mg/L, CEA ≥ 18.32 μg/L, tumor diameter ≥ 5.5 cm and palliative treatment after recurrence were independent risk factors for the prognosis of LR. Nomograms based on these risk factors showed good predictive power and could provide reference for clinicians’ treatment strategies to some extent.

## Author contributions

**Conceptualization:** Chenming Liu, Liang An.

**Data curation:** Liang An.

**Formal analysis:** Chenming Liu, Liang An.

**Funding acquisition:** Liang An.

**Investigation:** Liang An.

**Methodology:** Jialiang Lu, Liang An.

**Project administration:** Liang An.

**Resources:** Liang An.

**Software:** Liang An.

**Supervision:** Liang An.

**Validation:** Liang An.

**Visualization:** Liang An.

**Writing – original draft:** Chenming Liu.

**Writing – review & editing:** Liang An.

## Supplementary Material












