# Supplementary material for: Development and validation of nomograms for predicting the prognosis of early and late recurrence of advanced gastric cancer after radical surgery based on post-recurrence survival
Source: Medicine (Baltimore). 2024 May 31;103(22):e38376. doi: 10.1097/MD.0000000000038376 (PMC11142773; doi:10.1097/MD.0000000000038376)
Supplement: Supplementary file 1 [file medi-103-e38376-s001.docx]

**Table S1** The cut-off value for variables of early recurrence group by receiver operating characteristic.

| Variables | N | AUC | Sensitivity | Specificity | Youden index | Cut-off value |
| --- | --- | --- | --- | --- | --- | --- |
| Age | 67 | 0.601 | 0.593 | 0.625 | 0.218 | 68.0 |

| Variables | N | AUC | Sensitivity | Specificity | Youden index | Cut-off value |
| --- | --- | --- | --- | --- | --- | --- |
| PNI | 67 | 0.542 | 1.0 | 0.305 | 0.305 | 35.65 |

| Variables | N | AUC | Sensitivity | Specificity | Youden index | Cut-off value |
| --- | --- | --- | --- | --- | --- | --- |
| ALB | 67 | 0.55 | 0.75 | 0.424 | 0.174 | 32.5 |

| Variables | N | AUC | Sensitivity | Specificity | Youden index | Cut-off value |
| --- | --- | --- | --- | --- | --- | --- |
| PLB | 67 | 0.583 | 1.0 | 0.288 | 0.288 | 70.1 |

| Variables | N | AUC | Sensitivity | Specificity | Youden index | Cut-off value |
| --- | --- | --- | --- | --- | --- | --- |
| NLR | 67 | 0.695 | 0.661 | 0.75 | 0.411 | 2.018 |

| Variables | N | AUC | Sensitivity | Specificity | Youden index | Cut-off value |
| --- | --- | --- | --- | --- | --- | --- |
| LCR | 67 | 0.625 | 0.625 | 0.712 | 0.337 | 0.535 |

| Variables | N | AUC | Sensitivity | Specificity | Youden index | Cut-off value |
| --- | --- | --- | --- | --- | --- | --- |
| PLR | 67 | 0.595 | 0.949 | 0.375 | 0.324 | 71.681 |

| Variables | N | AUC | Sensitivity | Specificity | Youden index | Cut-off value |
| --- | --- | --- | --- | --- | --- | --- |
| LMR | 67 | 0.61 | 0.875 | 0.475 | 0.35 | 2.286 |

| Variables | N | AUC | Sensitivity | Specificity | Youden index | Cut-off value |
| --- | --- | --- | --- | --- | --- | --- |
| CEA | 67 | 0.689 | 0.678 | 0.75 | 0.428 | 4.4 |

| Variables | N | AUC | Sensitivity | Specificity | Youden index | Cut-off value |
| --- | --- | --- | --- | --- | --- | --- |
| CA199 | 67 | 0.629 | 0.525 | 0.875 | 0.4 | 25.52 |

| Variables | N | AUC | Sensitivity | Specificity | Youden index | Cut-off value |
| --- | --- | --- | --- | --- | --- | --- |
| Tumor size | 67 | 0.52 | 0.729 | 0.5 | 0.229 | 4.4 |

| Variables | N | AUC | Sensitivity | Specificity | Youden index | Cut-off value |
| --- | --- | --- | --- | --- | --- | --- |
| PLNR | 67 | 0.716 | 0.508 | 1.0 | 0.508 | 0.486 |
| PNI, prognostic nutrition index; ALB, albumin; PLB, prealbumin; NLR, neutrophil-to-lymphocyte ratio; LCR, lymphocyte-to-C-reactive ratio; PLR, platelet-to-lymphocyte ratio; LMR, lymphocyte-to-monocyte ratio; CEA, carcinoembryonic antigen; CA 199, carbohydrate antigen 199; PLNR, positive lymph nodes ratio. | | | | | | |
