# Supplementary material for: Development and validation of nomograms for predicting the prognosis of early and late recurrence of advanced gastric cancer after radical surgery based on post-recurrence survival
Source: Medicine (Baltimore). 2024 May 31;103(22):e38376. doi: 10.1097/MD.0000000000038376 (PMC11142773; doi:10.1097/MD.0000000000038376)
Supplement: Supplementary file 2 [file medi-103-e38376-s002.docx]

**Table S2** The cut-off value for variables of late recurrence group by receiver operating characteristic.

| Variables | N | AUC | Sensitivity | Specificity | Youden index | Cut-off value |
| --- | --- | --- | --- | --- | --- | --- |
| PLNR | 61 | 0.607 | 0.528 | 0.75 | 0.278 | 0.289 |

| Variables | N | AUC | Sensitivity | Specificity | Youden index | Cut-off value |
| --- | --- | --- | --- | --- | --- | --- |
| Age | 61 | 0.575 | 0.245 | 1.0 | 0.245 | 72.0 |

| Variables | N | AUC | Sensitivity | Specificity | Youden index | Cut-off value |
| --- | --- | --- | --- | --- | --- | --- |
| PNI | 61 | 0.708 | 0.75 | 0.679 | 0.429 | 44.0 |

| Variables | N | AUC | Sensitivity | Specificity | Youden index | Cut-off value |
| --- | --- | --- | --- | --- | --- | --- |
| Albumin | 61 | 0.732 | 0.75 | 0.698 | 0.448 | 37.7 |

| Variables | N | AUC | Sensitivity | Specificity | Youden index | Cut-off value |
| --- | --- | --- | --- | --- | --- | --- |
| Prealbumin | 61 | 0.724 | 0.875 | 0.585 | 0.46 | 170.1 |

| Variables | N | AUC | Sensitivity | Specificity | Youden index | Cut-off value |
| --- | --- | --- | --- | --- | --- | --- |
| NLR | 61 | 0.554 | 0.585 | 0.75 | 0.335 | 2.748 |

| Variables | N | AUC | Sensitivity | Specificity | Youden index | Cut-off value |
| --- | --- | --- | --- | --- | --- | --- |
| LCR | 61 | 0.651 | 0.625 | 0.792 | 0.417 | 1.729 |

| Variables | N | AUC | Sensitivity | Specificity | Youden index | Cut-off value |
| --- | --- | --- | --- | --- | --- | --- |
| PLR | 61 | 0.703 | 0.528 | 1.0 | 0.528 | 156.618 |

| Variables | N | AUC | Sensitivity | Specificity | Youden index | Cut-off value |
| --- | --- | --- | --- | --- | --- | --- |
| LMR | 61 | 0.596 | 0.625 | 0.679 | 0.304 | 3.073 |

| Variables | N | AUC | Sensitivity | Specificity | Youden index | Cut-off value |
| --- | --- | --- | --- | --- | --- | --- |
| CEA | 61 | 0.553 | 0.208 | 1.0 | 0.208 | 18.32 |

| Variables | N | AUC | Sensitivity | Specificity | Youden index | Cut-off value |
| --- | --- | --- | --- | --- | --- | --- |
| CA199 | 61 | 0.512 | 0.868 | 0.375 | 0.243 | 3.73 |

| Variables | N | AUC | Sensitivity | Specificity | Youden index | Cut-off value |
| --- | --- | --- | --- | --- | --- | --- |
| Tumor size | 61 | 0.723 | 0.528 | 0.875 | 0.403 | 5.5 |
| PNI, prognostic nutrition index; ALB, albumin; PLB, prealbumin; NLR, neutrophil-to-lymphocyte ratio; LCR, lymphocyte-to-C-reactive ratio; PLR, platelet-to-lymphocyte ratio; LMR, lymphocyte-to-monocyte ratio; CEA, carcinoembryonic antigen; CA 199, carbohydrate antigen 199; PLNR, positive lymph nodes ratio. | | | | | | |
