# Supplementary material for: Development and validation of nomograms for predicting the prognosis of early and late recurrence of advanced gastric cancer after radical surgery based on post-recurrence survival
Source: Medicine (Baltimore). 2024 May 31;103(22):e38376. doi: 10.1097/MD.0000000000038376 (PMC11142773; doi:10.1097/MD.0000000000038376)
Supplement: Supplementary file 3 [file medi-103-e38376-s003.docx]

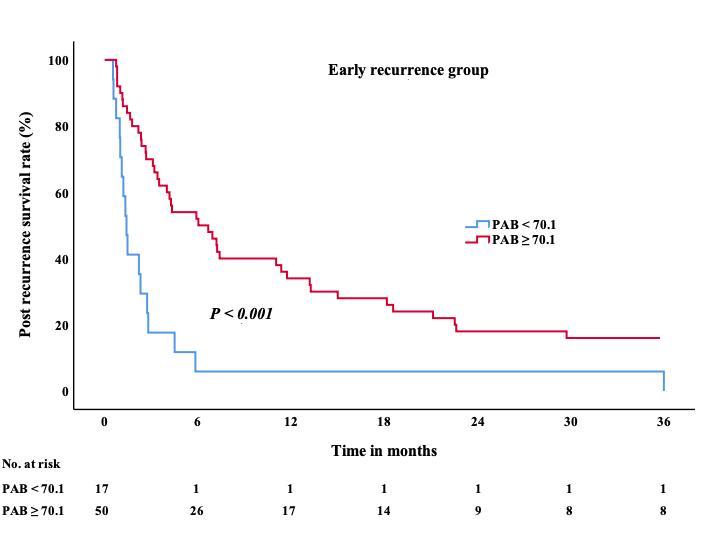


**Figure S1.** Kaplan-Meier curve of between different PAB levels for early recurrence group. PAB, prealbumin.
